# Effect of nano-composite and Thyme oil (Tymus Vulgaris L) coating on fruit quality of sweet cherry (Takdaneh Cv) during storage period

**DOI:** 10.1002/fsn3.226

**Published:** 2015-05-10

**Authors:** Naser Nabifarkhani, Mehdi Sharifani, Amir Daraei Garmakhany, Ebrahim Ganji Moghadam, Alireza Shakeri

**Affiliations:** 1 Department of Horticulture Science, Gorgan University of Agricultural sciences and Natural ResourcesGorgan, Iran; 2 Department of Food Science and Technology, Toyserkan Faculty of Industrial Engineering, Bu-Ali Sina UniversityHamadan, Iran; 3 Khorasan Razavi Natural Resource Agricultural Research CentreMashhad, Iran; 4 Faculty of Chemistry, Tehran UniversityTehran, Iran

**Keywords:** HPLC method, nano composite, storage periods, sweet cherry, thyme oilthyme oil

## Abstract

Sweet cherry is one of the most appreciated fruit by consumers since it is an early season fruit and has an excellent quality. In this study effect of active nano composite formed from chitosan (as a matrix material), nano cellulose fiber (1% concentration) and Thyme oils (*Tymus Vulgaris* L) at 1% concentration on fruits quality was investigated. Treated fruits were stored at 1°C for 5 weeks and changes of different qualities attributes including weight loss, total acidity, TSS, anthocyanin, total sugar and malic acid content (by high performance liquid chromatography (HPLC) method) were measured each week. Results showed that nano composite and Thyme oil significantly affect fruit's water retention and so decrease fruit weight loss and preserve anthocyanin (*P* < 0.05). None of applied treatments had any significant effects in comparison with control in regard to acidity while total sugar content and TSS significantly affected by treatment compared to control samples. Result of HPLC analysis showed that there was no significant difference between different treatment and control sample in term of malic acid concentrations during storage period but increase storage time lead to increase malic acid concentration in all treatments. For conclusion it can be Saied that fruits coating with nano-composite, lead to increase fruit shelf life, better appearance and prevents fungal growth that may be due to creation of an active packaging by these compounds.

## Introduction

Sweet cherry due to early season fruit ripening and excellent quality is one of the most popular fruit by consumers. The main quality indexes are skin colour, which is related to fruit ripening and affected by anthocyanin concentration (Serrano et al. 2005a), and the ratio of total soluble solids to total acidity (TSS/TA) at harvest time. Both parameters, together with the absence of stem browning determine consumer acceptance (Crisosto et al. 2003). TSS varied from 11 to 25 ^0^Brix, depending on cultivar and is mainly due to glucose and fructose and less to the presence of sucrose and sorbitol. In sweet cherry depending to the cultivar total acidity ranged between 0.4–1.5% and malic acid is the main organic acid (Esti et al. 2002; Bernalte et al. 2003). Colour is one of the most important indicators of maturity and quality of fresh, stored, and processed cherries fruit (Drake et al. 1982). In cherries, colour is mainly affected by the concentration and distribution of different anthocyanins in the skin (Gao and Mazza 1995). The major anthocyanins in sweet cherries include the 3-O-glucoside and 3-O-rutinoside (d-rhamnosyl d-gluco pyranose) of cyanidin, with peonidin-3-O-rutinoside and glucoside, as well as pelargonidin-3-O-rutinoside occurring in much lower amounts (Goncalves et al. 2004). Sweet cherry fruit deteriorate rapidly after harvest and in some cases do not reach consumers at optical quality after transport and marketing. Postharvest loss of quality in cherries is characterized by bruising of the skin, softening and loss of acidity (Bernalte et al. 2003), drying and browning of the stem (Kappel et al. 2002) and by fungal diseases caused mainly by *Monilinia fructicola*, *Botrytis cinerea* (Ganji Moghadam and Bosari 2009) and *Penicillium expansum*. This fungal spoilage can lead to great economic losses, although the occurrence of rots and their influence on sweet cherry quality have been reported to be dependent on cultivar and ripening stage at harvesting time (Esti et al. 2002; Kappel et al. 2002). Several pre and postharvest technologies have been used to control decay, but the postharvest use of chemicals as fungicides is restricted in most countries and consumers demand agricultural commodities without pesticide residues (Wilcock et al. 2004). Among these technologies, the use of edible coatings is traditionally used to improve food appearance and conservation. They act as barriers during processing, handling and storage, and do not solely retard food deterioration enhancing its quality, but are safe due to natural biocide activity, or to the incorporation of antimicrobial compounds (Petersen et al. 1999). Different compounds have mainly been used as edible coatings to prevent weight loss, including wax, milk proteins, celluloses derivatives, lipids, starch, zein, alginate, and chitosan (Cha and Chinnan 2004). Chitosan, obtained by deacetylation of chitin, might be an ideal preservative coating for fresh fruit because of its film-forming and biochemical properties (Muzzarelli 1986). Unlike other coating materials, chitosan is known to be antifungal to several fungi, including Botrytis cinerea And Rhizopus stolonifer (El Ghaouth et al., 1992), to induce chitinase, a defense enzyme (Mauch et al. 1984), and to elicit phytoalexin (pisatin) accumulation in pea (*Pisum sativum* L.) (Kendra and Hadwiger 1984). Furthermore, chitosan is a by-product from the seafood industry, appears to be a safe material as indicated by toxicological studies (Hirano et al. 1990). Since chitosan can form a semi permeable film (Bai et al. 1988), coating fruit with chitosan may modify the internal atmosphere of the tissue and consequently delay ripening. Essential oils are natural colorless compounds consisting of alcohols, aldehydes and esters that have fewer odors and its molecular weight is lower than water. Essential oils are volatile compounds that can be used as flavoring, antioxidant and antimicrobial agents in foods (Omid Beigi 2005). Also it can be used as edible coatings for fruits and vegetables to increase the shelf life of agricultural products (Park et al. 2002). According to their antioxidant role, they may be used to prevent enzymatic browning reaction (Nicoli et al. 1994). Some of the study reported the beneficial effects of natural essential oils on quality of different fruits including grapes, avocados and cherries (Serrano et al. 2005b). According to the introduction the aim of this study was investigation the alteration of quality attributes and reduction of decays in cherry fruit by use of edible coatings (nano composite) formed from chitosan and nano cellulose and Thyme oils (*Tymus Vulgaris* L) during storage periods.

## Materials and Methods

In this study more than 20 kg sweet cherry fruits from a commercial orchard located in Mashhad city of Iran was collected. Applied nano composite was made from chitosan (1% concentration) as the matrix material and nano-cellulose (0.1% concentration). Thyme oil was dissolved in ethanol 25% and used in 1% concentration. This project was conducted in three treatments; Control, nano composite, Thyme oil. Treated fruits were stored at a temperature of 1°C and 90% RH for 6 weeks and each week quality attributes of fruits were measured.

### Coating preparation

The method to prepare the coating solutions was developed by Alikhani et al. (2012) with minor modifications. The chitosan solution (1.0%) containing 0.5% acetic acid as a solvent was stirred by a magnetic stirrer at room temperature for 1 h to obtain complete dispersion. Then the thyme oil, mixed with tween 80 (0.2%), to help distribute and completely incorporate the thyme oil, was added to the chitosan solution and then stirred using a magnetic stirrer for 30 min. The final solution was centrifuged for 10 min at 2268 g and the supernatant obtained was used to prepare the edible coating.

### Total soluble solids (Brix) and titratable acid (mli eqi/g)

Total soluble solids (^0^BX) were determined according to AOAC (2005) using hand refrectometer at room temperature. Titratable acid was determined by titration of fruits juice by NaOH (0.1 N) until reach the pink color and express as the mli eqi of malic acid per gram of fruits samples.

### Fruit weight loss

Fruits weight loss for each treatment was calculated by the following equation at each storage periods: 

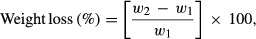
1where *w*_1_ is initial weight of fruit samples and *w*_2_ is Weight of fruit samples at each storage periods.

### Extraction and measurement of total sugar (mg/100 g of fresh tissue)

Total sugar was extracted using Omokolo et al. (1996) method. In this method, 40 mg of fresh fruits was taken and mixed with 5 mL of ethanol (80%) for 10 min in a hot water bath (70°C). The resulting extracts were centrifuged (1000 g) 15 min and obtained supernatant was transferred to a beaker, this process repeated 4 times on remained the residual tissue. The extract was concentrated by heating to 1/5 of its volume. The resulting aqueous phase was centrifuged (10000 g) for 10 and clear upper phase was removed and used for determination of soluble sugars.

Total soluble sugar was determined with McCready et al. (1950) method. For determination of total sugar, 0.2 mL (200 mL) from produced extract mix with 3 mL Antrun for 20 min in hot water bath (100°C). After cooling the absorbance of each sample was measured at 620 nm.

### Anthocyanin measurement (M/g of fresh tissue)

The amount of anthocyanin was measured by Wanger (1979) method. For determination of anthocyanin 1 g of fresh cherry fruit juice was taken and mixed with 10 mL acidified methanol and kept in the dark place (4°C) for 24 h. Then, the extract was centrifuged (4000 g) for 10 min and absorbance of the supernatant was read in 520 nm using a spectrophotometer (S 2000 uv/vis).

### Malic acid measurement by HPLC method

Malic acid was quantified by high performance liquid chromatography (HPLC). The analysis was carried out using a Shimadzu chromatograph, model LC-10 Ai (Shimadzu Corp., Kyoto, Japan), equipped with an Ultra Violet detector (UV- SPD-6AV). A Shimadzu column C18 (Shim-pack SCR-101H, separating column 7.9 mm×30 cm), was operated at 25°C for organic acids. Mobile phase was consisted of isocratic and acidic dionized water with sulfuric acid (pH = 2) at a flow rate of 1 mL/min. The malic acid content of the samples was detected via PID detector (214 nm). The quantification of malic acid was performed using calibration curve obtained from the standard compound. All samples were examined in duplicate. The sensitivity of system was equal 3 and the temperature of separating column was 25°C. The injection was carried out using 20 *μ*L volume of the sample with application of Hamilton syringe. Before injection the samples passed through the filter with pores diameter of 0.45 *μ*. The concentration of malic acid calculated using standard curve, retention time and the space under the curve of each sample.

### Statistical analysis

This experiment was conducted in a completely randomized factorial design. The first factor consists of edible coatings: control, nano composite, and thyme oil respectively. The second factor is the storage time (0, 7, 14, 21, 28 and 35 days of storage) respectively. Results of this study were analyzed using SAS (2001) software and means comparison was performed by Duncan's multiple range test (95%). All the experiments and treatment were done with three replications.

## Results and Discussion

### Titratable acid

**Figure 1:**
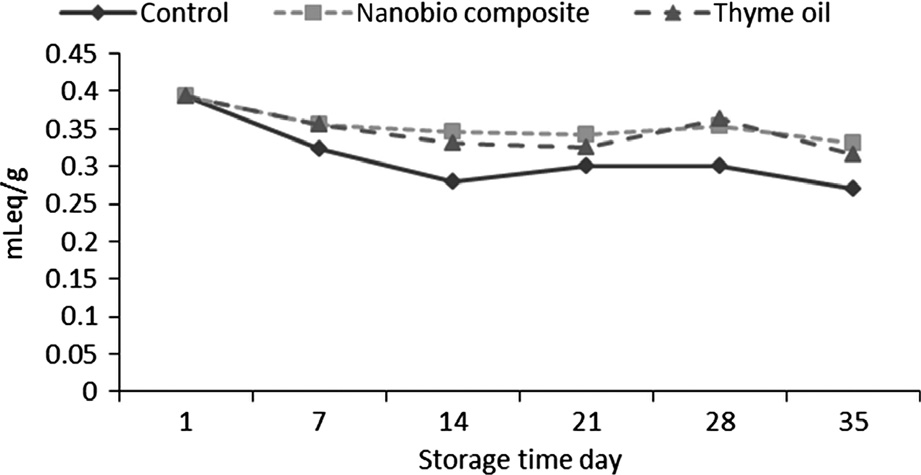
Effect of time and coating on titratable acid content in cherry fruit.

Based on the results (Fig.1), the total acidity decreased during the storage period. Nano composite and thyme oil lead to preserve organic acids in cherry fruit significantly. Reduction of respiratory rate, lead to reduction of the amount of organic acids consumption. Finidokht et al. (2013) showed that chitosan by decrease respiration, reduces the amount of organic acids consumption in cherry fruit, while increase storage time lead to more consume organic acids. They stated that the chitosan in short time storage periods preserve organic acids while in long time storage periods their protection properties will decrease. Previous studies showed that chitosan had positive effect on organic acids perseveration in tomato and strawberry fruit (Kittur et al., 1998; Lydakis and Aked 2003).

### Malic acid analysis using HPLC

**Figure 2:**
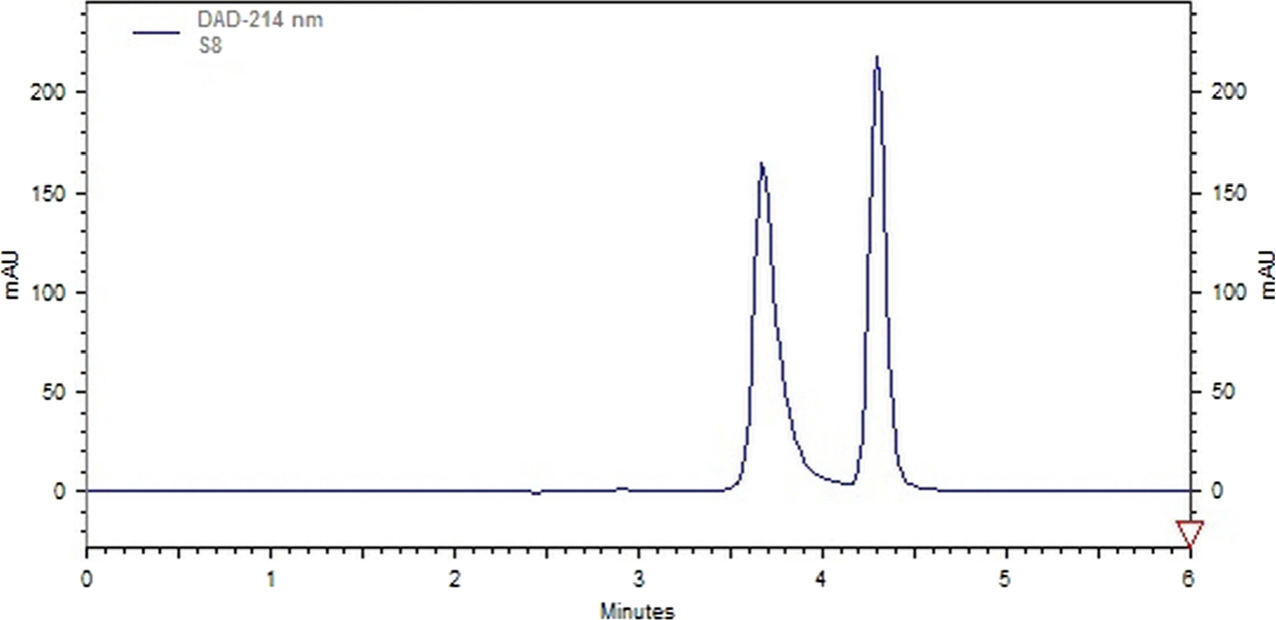
Typical HPLC curve for malic acid determination.

Results of malic acid analysis using HPLC method showed that the concentration of malic acid in control samples was preserved with an increasing trend from 1st week to 3rd of storage periods (Fig.2). In the first week of storage the malic acid concentration was 4.9 gr/lit and in 3rd week of storage the concentration increased to 6.76 g/lit. However the malic acid concentration at 6th week was 7.16 g/lit.

In 3rd week of storage period, application of essential oil treatment caused a concentration of malic acid reached to 5.67 g/lit and in the 6th week of storage period malic acid concentration was 6.87 g/lit. This result revealed the effect of essential oil on preservation of fruit water content. Higher malic acid concentration was due to the less evaporation rate from fruit surface by using essential oil treatment. There was no difference between control and nano-composite treatment in term of malic acid concentrations until 3rd week of storage. The concentration of malic acid for nano-composite and control treatment was 7.3 and 7.16 g/lit in 6th week of storage respectively.

The final conclusion of malic acid analysis among the applied treatments indicated that 3 weeks of storage is the most acceptable storage period for essential oil and nano composite treatments, whereas there was no significant difference between applied treatments in term of of malic acid concentration during storage period. It might be offered that malic acid concentration is not a useful criterion to judge the efficiency of treatments. Water status of fruit, turgor pressure, antioxidant activity, sugar and anthcyanin content are other crucial criteria's which affect judgment on efficiency of the treatments.

### Weight loss, total soluble solid (TSS) and soluble sugar

**Figure 3:**
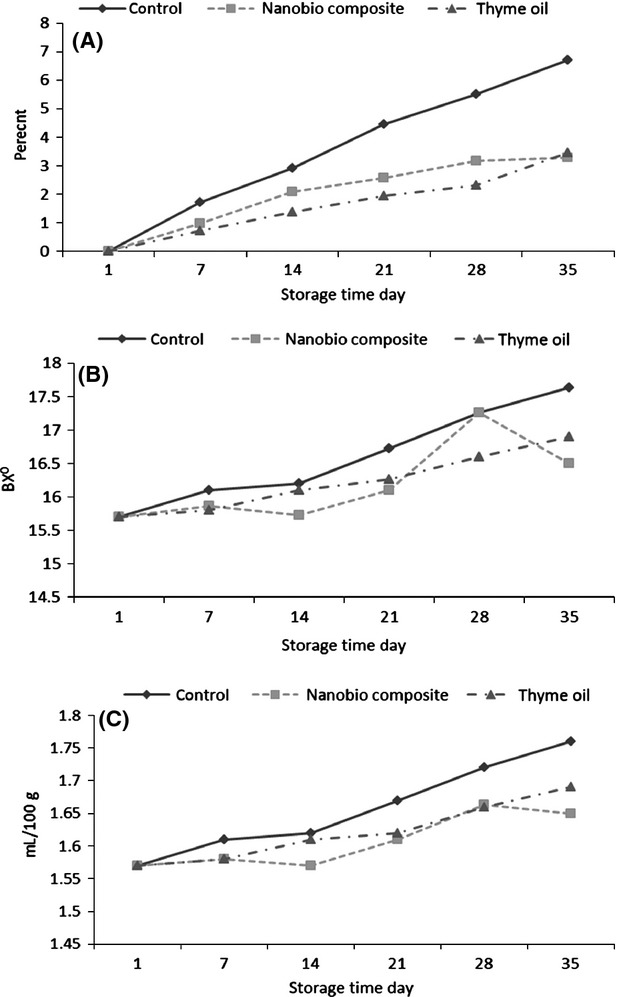
Effect of time and coating on (A) weight loss (B) total soluble solid and (C) soluble sugar content in cherry fruit.

Based on the results (Fig.3A) the greatest weight loss was related to control samples and nano composite and thyme oil showed lower trend in weight loss than control samples which is in agreement with the results of Finidokht et al. (2013) and Shamloo et al. (2013). They stated that coated cherries with chitosan had lower water loss than uncoated cherries. It can be concluded that nano composite and thyme oil treatments by creation a physical barrier against moisture loss from fruits skin, prevent surface dehydration, fruits shrinkage and decrease respiration during storage. Also the higher water loss in uncoated Cherry than coated ones was due to an increase in respiration and evaporation (Finidokht et al. 2013). Further research for example; Ebrahimpur Komeleh et al. (2008) reported that Hindi clove oil at concentration of 500 ppm, lead to the lowest percentage of weight loss while control samples (uncoated) and treated samples by cumin and clove oil fumigation has the greater weight loss.

Results showed that TSS gradually increased during storage (Fig.3B) that may be due to activity of hydrolytic enzymes or increase of water loss during storage period (Alonso et al. 2003). At the end of storage period, the lowest (16.5 ^0^BX) and highest (17.63^0^BX) amount of TSS was related to nano composite and control samples respectively. Type of coating agent and storage time had significant effect on amount of fruit TSS during storage (Table1). Our result in term of TSS was in agreement with other studies (Vesal talab and Gholami; 2012). They study the gradual increase in the amount of TSS during storage of grape fruit treated with clove oil and clove extract compared to control.

**Table 1:** ANOVA of weight loss, TA, TSS, Total sugar and anthocyanin in sweet cherry as a function of storage time and coating type.

Surce of variation	Df	Weight loss (%)	TA (mleq/g)	TSS (%)	Total sugar (mg/100 g)	Anthocyanin (M/g)
Type of cover	2	14.3**	0.004*	1.28*	0.01*	0.454**
Time	5	27.98**	0.009**	3.05**	0.03**	6.35**
Type of cover × time	10	1.33**	0.003^ns^	0.109^ns^	0.01^ns^	0.119*

Superscripts ns, ^*^ and ^**^ means not significant, significant at 0.05 and 0.01 level respectively.

As can be seen from Figure3C, nano composite and thyme oil treated samples had lower content of soluble sugar than control sample. Total sugar content increased during storage period that may be due to the dehydration and decomposition of organic acids (used as an energy source) in the fruit (vesal talab and Gholami; 2012).

### Anthocyanin

**Figure 4:**
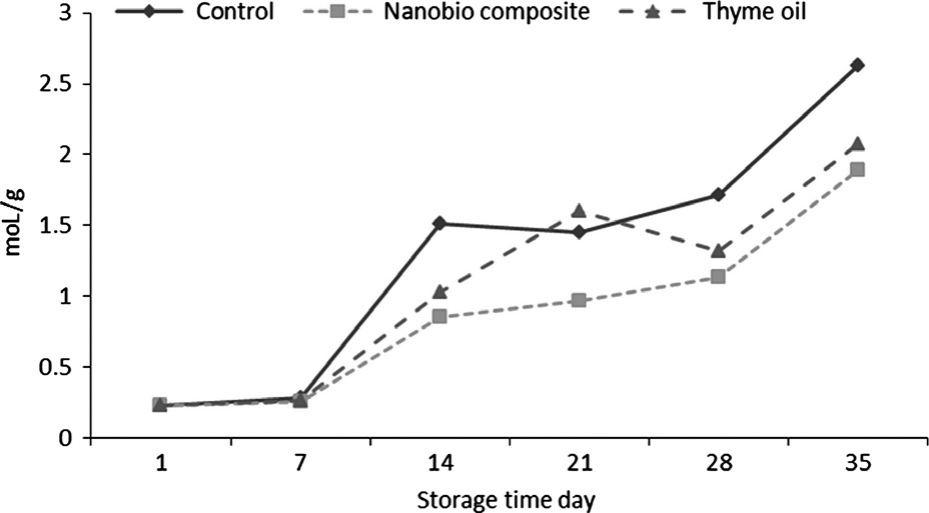
Effect of time and coating on antocyanin content of cherry fruit.

As can be seen in Figure4, anthocyanins increase during storage period. At the end of storage period, the lowest (1.89 mol/g) and highest (2.63 mol/g) amount of anthocyanin was related to nano composite and control samples respectively. Anthocyanin results were in agreement with the results of Shoja et al. (2011). They showed that anthocyanins increases during storage in blood orange.

## Conclusions

Applied treatments (nano composite and thyme oil) lead to preserve TSS, anthocyanins and total sugar content and reduce fruit weight loss in comparison with control samples (*P* < 0.05) while there are no significant differences between applied treatments (nano composite and thyme oil) and control in regard to titrable acidity and malic acid concentration. Results of this study showed that application of edible coatings can be used to increase fruit shelf life, better appearance of fruits and prevents fungi growth that may be due to creation a sort of active packaging by these compounds.

## Conflict of Interest

None declared.
